# Exploring Bias in Medical Applications of Large Language Models: Protocol for a Systematic Review

**DOI:** 10.2196/91348

**Published:** 2026-09-10

**Authors:** Shweta Rao, Carol Boutrous, Andrey Kormilitzin, Xin You Tai, Lewis Hotchkiss, Cen Cong, Edward Meinert, Huizhi Liang, Hang Dong, Judith R Harrison

**Affiliations:** 1 School of Medicine, Newcastle University, Newcastle, AK, United Kingdom; 2 Department of Psychiatry, University of Oxford, Headington, Oxford, England, United Kingdom; 3 Nuffield Department of Clinical Neurosciences, University of Oxford, Oxford, England, United Kingdom; 4 Dementias Platform UK (DPUK), Swansea University, Swansea, Wales, United Kingdom; 5 Translational and Clinical Research Institute, Faculty of Medical Sciences, Newcastle University, Newcastle, England, United Kingdom; 6 Translational and Clinical Research Institute 3rd Floor, Biomedical Research Building Campus for Ageing and Vitality, Westgate Road, Newcastle upon Tyne NE4 5PL, Newcastle, England, United Kingdom, 44 7756339941; 7 School of Computing, Newcastle University, Urban Sciences Building 1, Science Square, Newcastle upon Tyne, United Kingdom; 8 Department of Computer Science, University of Exeter, Exeter, United Kingdom

**Keywords:** large language model, LLM, bias, health care, medicine

## Abstract

**Background:**

Large language models (LLMs) are increasingly applied in health care for clinical decision-making, education, and patient communication. However, bias in LLM outputs may exacerbate health care disparities and compromise trust. Despite rapid adoption, there is limited synthesis of how bias is identified, measured, and mitigated in medical applications of LLMs.

**Objective:**

This systematic review aims to evaluate how bias is detected, measured, and mitigated in health care applications of LLMs and to identify methodological trends and gaps in the current literature.

**Methods:**

We will conduct a systematic review of studies evaluating bias, fairness, or subgroup performance in medical applications of LLMs. Searches will be conducted across Embase, MEDLINE, PsycINFO, PubMed, ACL Anthology, ACM Digital Library, arXiv, medRxiv, and bioRxiv for studies published from 2017 onward. The review uses a staged design. In phase 1, records identified in the original June 2025 search were screened and assessed manually using predefined eligibility criteria. This fully manually reviewed dataset will serve as a reference standard for validating the review workflow. In phase 2, an updated June 2026 search will be screened using a prespecified LLM-assisted workflow. Two independent LLMs will apply the same eligibility criteria used in manual screening, with studies marked as potentially relevant or uncertain by either model progressing to further assessment. Final inclusion decisions will be made by at least 2 human reviewers. The performance of the LLM-assisted workflow will be evaluated against the manually reviewed reference dataset using sensitivity, specificity, precision, negative predictive value, inclusion agreement, and Cohen κ. Data will be extracted using a standardized framework covering study characteristics, model details, health care use case, bias type, bias assessment methods, mitigation strategies, transparency, generalizability, and ethical or regulatory framing. Findings will be synthesized narratively.

**Results:**

The phase 1 search, conducted on June 11, 2025, yielded 15,976 records after deduplication and has undergone complete manual screening. The phase 2 search, conducted on June 8, 2026, increased the total number of records to 41,143. Screening of the updated dataset using the LLM-assisted workflow is ongoing. The completed review will report included study characteristics, approaches to bias detection and mitigation, and validation metrics for the LLM-assisted workflow. The review is expected to be submitted for publication in late November 2026, with publication anticipated thereafter subject to the journal’s peer-review and editorial process.

**Conclusions:**

This review aims to provide a comprehensive synthesis of current approaches to bias detection and mitigation in medical LLMs, highlighting methodological strengths, limitations, and areas for future research. The findings aim to inform the development of more equitable and transparent AI systems in health care.

## Introduction

Large language models (LLMs) are advanced transformer-based AI systems trained on massive volumes of text data, enabling them to generate fluent, contextually appropriate language. Recent innovations in LLM architectures, including Generative Pretrained Transformer [1], Bidirectional Encoder Representations from Transformers [2], Pathways Language Model [3], and Large Language Model Meta AI [4], have significantly expanded the scope of these models. In medicine, LLMs are increasingly applied to tasks such as clinical decision support [5], automated documentation [6], patient communication [7], and biomedical literature analysis [8], offering the potential to improve efficiency, accessibility, and patient engagement.

Unlike traditional rule-based systems, LLMs can synthesize unstructured medical data, respond dynamically in conversational contexts, and assist with real-time medical information retrieval [9]. Their capacity to model nuanced language use makes them promising tools for augmenting health care delivery and education. However, this flexibility also introduces substantial risk: LLMs are inherently probabilistic and operate through high-dimensional latent representations and self-attention mechanisms [10]. As a result, their outputs are nondeterministic, opaque, and potentially inconsistent [11], posing challenges for transparency, verifiability, and safety in clinical settings [6,12,13].

Bias in LLMs refers to systematic errors in output that arise from imbalances in training data, annotation processes, or architectural constraints [14]. In this review, bias refers specifically to systematic differences in model performance or outputs across demographic or clinically relevant subgroups. This is conceptually distinct from related phenomena such as hallucination, which refers to the generation of factually incorrect or unsupported information [15], and from broader considerations relating to AI safety, accountability, and transparency. While these issues may co-occur in practice, this review focuses on bias as it relates to systematic inequities in outputs.

In health care, such biases can manifest as disparities in diagnostic accuracy, therapeutic recommendations, or patient communication. For instance, if training data overrepresent certain populations or reflect historically biased clinical guidelines, LLM outputs may reinforce these inequities. Notable examples include race-based estimations in kidney function [16] or gender disparities in cardiovascular diagnosis and treatment [17], biases that LLMs may inherit and perpetuate if not explicitly addressed.

The complexity of LLMs compounds the difficulty of bias detection and mitigation. Because these models generate outputs without a fixed decision pathway, biases may appear subtly and variably, eluding traditional evaluation metrics [18]. Techniques such as dataset audits, counterfactual fairness testing, and adversarial evaluation have emerged to assess and quantify bias, yet no unified framework exists for their systematic application in health care LLMs [18-20]. Moreover, while some mitigation strategies, such as data augmentation, algorithmic debiasing, and retrieval-augmented generation (RAG) [21-23], have been proposed, their real-world effectiveness remains uncertain, and in some contexts, these approaches may also introduce or exacerbate bias.

Recent systematic reviews have begun to examine aspects of bias and fairness in medical LLMs, including demographic disparities in model performance [24]. However, these studies primarily focus on identifying and describing bias rather than systematically evaluating the methodologies used to detect and address it. Other reviews have either focused on classical machine learning or deep learning in medical imaging and disease diagnosis [25-32] or have surveyed LLM applications in health care without a primary focus on fairness or bias [33]. As the integration of LLMs into health care accelerates, there is a growing need to systematically evaluate how bias is identified, measured, and mitigated. This review aims to address this gap by synthesizing current approaches across medical applications. It focuses specifically on LLM uses with direct or potential near-term clinical relevance to ensure that findings are applicable to real-world health care settings.

The rapid growth of the literature on medical LLMs presents challenges for evidence synthesis, particularly where large numbers of records require screening and assessment. Recent studies have explored the use of LLMs to support systematic reviews, demonstrating improved efficiency while maintaining high agreement with human reviewers [34]. Such approaches may provide a scalable means of managing increasing volumes of evidence while preserving methodological rigor through appropriate validation procedures.

## Methods

### Objectives

This systematic review aims to identify and evaluate how bias is detected, measured, and mitigated in applications of LLMs within medical contexts. Specifically, it will

1. map the medical use cases of LLMs and determine in which domains bias has been evaluated (eg, clinical decision support, documentation, patient communication, education, or research).
2. assess the methodologies employed for bias detection and measurement, including dataset audits, counterfactual fairness testing, adversarial evaluation, and explainability techniques.
3. catalog the bias mitigation strategies reported in the literature, such as data augmentation, algorithmic debiasing, RAG, and post hoc correction techniques.
4. synthesize the evidence into preliminary best practice guidance, highlighting effective approaches for bias assessment and mitigation to inform future research, development, and regulatory oversight of LLMs in health care.

### Research Questions

To ensure a structured approach to defining the scope of this systematic review, we adopted the Population-Concept-Context (PCC) framework [35] for systematic reviews that aim to map methodologies rather than evaluate interventions. In this review:

- Population (P): LLMs applied to health care.
- Concept (C): Bias detection methods and mitigation strategies.
- Context (C): Clinical, educational, and research applications of LLMs in medicine.

Based on this framework, our review addresses the following research questions:

- What medical applications use LLMs, and in which of these has bias been assessed?
- What methods have been used to detect and measure bias in medical LLMs, including those related to demographic disparities (eg, race, gender, age), clinical misrepresentation, or dataset imbalances?
- What mitigation strategies have been implemented to reduce bias in medical LLMs?
- To what extent do current studies report on the effectiveness or limitations of these bias mitigation approaches?
- Based on existing evidence, what best practices can be recommended for future research on bias assessment and mitigation in medical LLMs?

### Eligibility Criteria

For the purposes of this review, LLMs are broadly defined as transformer-based natural language processing models applied to health care, including encoder-only, decoder-only, and encoder-decoder architectures. Encoder-only models (eg, BERT variants) were included because they remain widely used in health care fairness and subgroup-performance research. Multimodal or foundation models incorporating vision-language or speech-language capabilities will be included only where language generation or language-based clinical decision support forms a substantial component of the evaluated system.

The following inclusion criteria were established to ensure that selected studies directly investigate bias in medical LLM applications and provide empirical findings on detection or mitigation strategies:

1. Studies must involve an LLM or foundation language model applied to a medical or health care context and assess bias, fairness, or subgroup performance in model outputs.
2. Eligible studies include experimental, simulation, benchmarking, audit, or evaluation studies using real-world or synthetic datasets, provided they assess bias, fairness, or subgroup performance within a clinically relevant health care context.
3. The study must be published in English.
4. Papers published from 2017 onward, corresponding to the publication of the Transformer architecture [36], which forms the foundational basis of contemporary LLMs.
5. Studies examining training or fine-tuning will be included only if they evaluate model outputs, performance, or bias in a clinically relevant context.
6. Gray literature (eg, preprints, conference proceedings, technical or institutional reports) will be included if sufficient methodological detail, clear data sources, and reported findings relevant to bias or fairness are provided.

The following exclusion criteria were defined to remove studies that do not focus on bias in medical LLMs, lack empirical evaluation, or investigate nonmedical AI applications:

1. Studies focusing solely on machine learning models or algorithms other than LLMs.
2. Reviews, editorials, letters, commentaries, opinion pieces, magazine articles, or abstract-only conference records without original empirical data.
3. Studies focusing solely on bias in word embedding models, such as Word2Vec, without examining LLMs or foundation language models.
4. Studies focused solely on pretraining or fine-tuning without subsequent evaluation of model outputs, performance, or bias in a medical or health care context.
5. Studies discussing ethics, hallucination, transparency, or safety without assessing bias, fairness, or subgroup performance.
6. Studies conducted solely in medical education settings without clear clinical relevance.
7. Gray literature lacking sufficient methodological detail, clear data sources, or identifiable authorship or institutional credibility. Abstract-only records will also be excluded.

### Review and Design Workflow

This review uses a staged design to enable comprehensive evidence synthesis in a rapidly expanding research field. The original search, completed in June 2025, was screened and assessed manually using predefined eligibility criteria. This phase provides a fully manually reviewed reference-standard dataset. An updated search, completed in June 2026 across the same information sources, will be used to identify additional eligible studies published since the original search.

To manage the expanded evidence base while maintaining methodological rigor, the updated search will be screened using a prespecified LLM-assisted workflow. The LLM-assisted workflow will apply the same eligibility criteria as the manual review and will be validated against the manually reviewed reference-standard dataset before interpretation of the updated search results. Studies identified as potentially eligible or uncertain by either LLM will proceed to further assessment, and final inclusion decisions will be made by at least 2 human reviewers.

This staged approach allows the review to retain the reliability of a completed manual review while also evaluating a scalable workflow for updating evidence synthesis in a fast-moving area of medical AI research. Any amendments to this protocol will be documented with a rationale and date and updated in the PROSPERO (International Prospective Register of Systematic Reviews) record.

### Search Methods

This protocol follows the PRISMA-P (Preferred Reporting Items for Systematic Reviews and Meta-Analyses Protocols) [37] checklist to ensure methodological transparency and reproducibility. The PRISMA-P guidelines inform the study selection process, search strategy development, and reporting structure.

### Confirmation of No Other Registered Reviews on This Topic

To confirm the originality of this review and prevent duplication, we searched for registered but incomplete or unpublished systematic and scoping reviews on this topic. The following databases were examined:

- CENTRAL (Cochrane Central Register of Controlled Trials)
- PROSPERO
- Evidence-Based Health Database (Epistemonikos)

No ongoing or unpublished reviews specifically addressing bias detection and mitigation in medical LLMs were identified.

### Information Sources and Search Strategy

We conducted a comprehensive search of electronic databases, including Embase, MEDLINE, PsycINFO, PubMed, ACL Anthology, ACM Digital Library, arXiv, medRxiv, and bioRxiv. In addition to biomedical databases, we included ACL Anthology and ACM Digital Library to capture relevant research from computational linguistics and AI development, as LLM bias often originates from model architecture and training practices.

Search terms included a combination of MeSH and free-text keywords related to LLMs and bias in medical applications. MeSH terms were applied where available (eg, *“artificial intelligence*” AND *“Bias in health care*”), while free-text keywords (eg, *“large language model bias*,” *“GPT in medicine*,” *“AI fairness in health care*”) were used to ensure comprehensive retrieval across databases that do not support MeSH.

A broad search strategy was employed to maximize sensitivity and ensure that relevant studies were not missed. This included the use of general terms (eg, accuracy, ethics, hallucination) that may overlap with bias-related concepts. Specific inclusion criteria and screening procedures were applied to ensure that only studies directly assessing bias, fairness, or subgroup performance are included. This approach reflects the evolving and inconsistently defined nature of bias in LLM research, where relevant concepts may be described using varied terminology across disciplines.

The search strategy was refined through pilot searches and consultation with an experienced health sciences librarian from Newcastle University. Boolean operators, truncation, and wildcards were applied where appropriate to optimize retrieval. Additional search techniques included backward and forward citation searching. Reference lists of included studies were screened for additional articles, and studies citing included papers were identified using Scopus, Web of Science, and Google Scholar. Studies identified through citation searching underwent the same eligibility screening process as database search results. Full search strategies for all databases, including exact query strings, applied filters, and record counts for final searches, are provided in Multimedia Appendices 1 and 2.

The search elements are shown in Table 1.

**Table 1.** Search terms.

Search element	Concept	Synonyms and keywords	MeSH terms (if applicable)
Population (P)	LLMs^a^	LLM*^a^ OR LMM* OR MLLM OR “Vision-Language Models” OR “Audio-Language Models” OR “Speech Language Models” OR LVLM OR VLM OR GPT*^b^ OR BERT^c^ OR LaMDA^d^ OR PaLM^e^ OR LLaMA^f^ OR Claude OR Alpaca OR Falcon OR BLOOM OR “Generative AI” OR “Transformer-based model*” OR “Foundational model*” OR OPT^g^ OR Fairseq^h^ OR Deepseek OR Gemini OR Med-PaLM OR Radiology-LLaMA	“Natural Language Processing”[MeSH] OR “Artificial Intelligence”[MeSH]
Context (C)	Health care and medical applications	Health* OR Medic* OR Clinic* OR Patient* OR EHR^i^ OR Physician* OR “Clinical Decision Support” OR “Medical AI” OR “Biomedical NLP^j^” OR “Biomedical Natural Language Processing” OR BioNLP^k^	“Health”[MeSH] OR “Electronic Health Records”[MeSH] OR “Clinical Decision Support Systems”[MeSH] OR “Health Services”[MeSH]
Concept (C)	Bias, fairness, ethics	Bias* OR Prejudice* OR Accuracy OR Ethic* OR Hallucination* OR Fair* OR Discrimination OR Responsible OR “Human Value Alignment” OR Inequity OR Disparit* OR Equalit* OR “Algorithmic Bias” OR “Explainability” OR “Transparency” OR “Accountability”	“Bias (Epidemiology)”[MeSH] OR “Health Equity”[MeSH] OR “Ethics, Medical”[MeSH] OR “Social Discrimination”[MeSH]

a LLM: large language model.

b GPT: Generative Pretrained Transformer.

c BERT: Bidirectional Encoder Representations from Transformers.

d LaMDA: Language Model for Dialogue Applications.

e PaLM: Pathways Language Model.

f LLaMA: Large Language Model Meta AI.

g OPT: Open Pretrained Transformer.

h FAIRSEQ: Facebook AI Research Sequence-to-Sequence Toolkit.

i EHR: electronic health record.

j NLP: natural language processing.

k BioNLP: biomedical natural language processing.

### Study Screening and Selection

All records identified through the original June 2025 search were imported into Rayyan and deduplicated. Titles and abstracts were screened manually by members of the review team using predefined eligibility criteria. Records considered potentially eligible underwent full-text assessment. Reasons for exclusion at the full-text stage were recorded using predefined categories. Disagreements were resolved through discussion, with consultation from an additional reviewer where required.

The manually reviewed June 2025 dataset will be used as the reference standard for validating the LLM-assisted screening workflow. Records identified through the updated June 2026 search will be screened using the LLM-assisted workflow described below, followed by human verification of potentially eligible studies. The study selection process will be reported using a PRISMA (Preferred Reporting Items for Systematic Reviews and Meta-Analyses) 2020 flow diagram.

### LLM-Assisted Screening Workflow

The LLM-assisted screening workflow is being finalized and will be specified before application to the updated search results. The final workflow will document the LLMs used, model versions, access dates, prompting strategy, input fields, classification labels, decision thresholds, and rules for resolving uncertain outputs. Two independent LLMs will screen records using the same eligibility criteria applied in the manual review. At the title and abstract stage, records will be classified as potentially eligible, excluded, or uncertain. Records classified as potentially eligible or uncertain by either LLM will progress to further assessment. Records will not be excluded on title alone.

Before interpretation of the updated search results, the LLM-assisted workflow will be evaluated against the manually reviewed June 2025 reference-standard dataset. Sensitivity will be treated as the primary screening-safety metric, because missed eligible studies represent the principal risk of an assisted screening workflow. Specificity, precision, negative predictive value, inclusion agreement, and Cohen κ will be reported as secondary metrics. Disagreements between manual and LLM-assisted decisions will be reviewed to identify potential sources of error. A sample of records excluded by the LLM-assisted workflow will undergo human audit to assess the risk of missed eligible studies, with particular attention to records using ambiguous terminology or reporting bias, fairness, or subgroup performance indirectly.

All records identified as potentially eligible through the LLM-assisted workflow will undergo final assessment by at least 2 human reviewers before inclusion. The completed systematic review will report the final LLM-assisted workflow in full, including model details, prompt templates, validation results, and any workflow modifications made before screening the updated search results.

### Data Extraction

A standardized data extraction form was developed and piloted on a subset of studies to ensure clarity and consistency (Multimedia Appendix 3). Data extraction has been completed for studies included in the manual review using this form. For studies identified through the ongoing LLM-assisted workflow, data extraction will be conducted using an LLM-assisted approach based on the same predefined framework to ensure consistency across review pathways.

Any discrepancies identified during data extraction will be resolved through discussion, with involvement of an additional reviewer where necessary to achieve consensus. Extracted data will be entered into a structured spreadsheet hosted within a secure, version-controlled repository accessible to members of the review team.

Upon completion of the review, a deidentified and finalized version of the dataset will be made available through an open-access repository (eg, OSF or Zenodo) to support transparency, reproducibility, and future reuse.

### Data Items

The data to be extracted are shown in Table 2.

**Table 2.** Data extraction categories and items.

Category	Data
Study characteristics	Title, publication date, journal, country of corresponding author, study type, source of funding, conflict of interest, primary objective
Study design	Study type (eg, experimental, observational, simulation, benchmarking), comparator(s) used (eg, human, ML^a^, other LLM^b^), evaluation date
LLM model details	Model name and version (eg, GPT^c^-3.5, GPT-4 March 2023), parameter count, hyperparameters (eg, temperature, top-p), domain specificity, fine-tuning, and prompt engineering will be extracted where reported. Where such details are unavailable, they will be recorded as “not reported” and will not be used as exclusion criteria.
Training data context	Reported sources of training data (eg, PubMed, MIMIC-III^d^), whether general or specialized corpus
Medical applications	Specific medical, clinical, educational, or patient-facing tasks (eg, diagnosis, consultation, summarization)
Bias assessment methods	Bias detection methods, fairness metrics, demographic groups evaluated, impact of bias (effects on clinical reasoning, decision quality, user trust, or health care disparities)
Bias mitigation strategies	Methods used to reduce bias (eg, algorithmic debiasing, adversarial training, RAG^e^), effectiveness of mitigation
Transparency	Explainability, prompt disclosure, justification for model outputs, external validation, and transparency reporting where available
Ethical considerations	Ethical principles discussed (eg, fairness, safety, autonomy), potential harms identified, references to formal or regulatory frameworks, recommendations for responsible AI

a ML: machine learning.

b LLM: large language model.

c GPT: Generative Pretrained Transformer.

d MIMIC-III: Medical Information Mart for Intensive Care (version III).

e RAG: retrieval-augmented generation.

### Assessment of Meta-Biases

Given the heterogeneity of study designs and outcomes anticipated in this review, quantitative assessment of publication bias (eg, funnel plots or Egger test) is unlikely to be appropriate. Instead, potential meta-biases will be assessed qualitatively. This will include comparison of findings from peer-reviewed publications and preprints, examination of selective outcome reporting (eg, incomplete reporting of subgroup or fairness analyses), and assessment of transparency in methodological reporting. The presence and potential impact of publication and reporting bias will be considered when interpreting the strength and consistency of the evidence.

### Confidence in Cumulative Evidence

Formal grading of evidence using GRADE (Grading of Recommendations Assessment, Development and Evaluation) is not well suited to the objectives of this review, which focuses on mapping methodological approaches to bias detection and mitigation in medical LLMs rather than estimating intervention effects. Instead, confidence in the cumulative evidence will be assessed qualitatively based on study design, methodological transparency, robustness of bias assessment methods, consistency of findings across models and application domains, and the tailored study quality assessment framework described in this protocol. This approach aligns with guidance for evidence synthesis in emerging and methodologically heterogeneous fields.

### Synthesis and Presentation of Results

The review will adhere to PRISMA guidelines, with a flow diagram detailing study selection and exclusion at each stage. Extracted data will be systematically analyzed and synthesized using a narrative approach, as the heterogeneity of study designs and methodologies precludes meta-analysis. A thematic synthesis approach will be applied, involving coding of extracted data, development of descriptive themes, and generation of analytical themes across studies. The synthesis will focus on patterns in bias detection methods, mitigation strategies, and their reported effectiveness. Ethical and governance themes identified across included studies will be incorporated into the narrative synthesis to contextualize methodological and clinical implications of bias in health care LLM applications. Studies will be grouped by health care use case (eg, clinical note summarization, diagnostic categorization), bias type (eg, demographic, clinical, systemic), bias detection methods (eg, dataset audits, counterfactual testing, adversarial evaluation), and mitigation strategies (eg, debiasing algorithms, RAG, dataset augmentation). Visual summaries, such as bar charts or heatmaps, may be used to illustrate the distribution of bias types and methodological approaches across studies.

### Measures to Minimize Bias in the Review Process

The systematic review protocol includes measures to reduce selection bias, such as transparent documentation of search strategies, inclusion criteria, and data extraction methods. Any potential conflicts of interest among reviewers will be declared, and efforts will be made to ensure objectivity.

- Reviewer selection: A diverse group of reviewers with varying academic and professional backgrounds will be involved in study selection, data extraction, and synthesis to minimize bias.
- Validation framework: The LLM-assisted workflow will be evaluated against a manually reviewed reference dataset. Studies identified as eligible through the LLM-assisted workflow will undergo independent review by at least 2 human reviewers before final inclusion.
- Agreement assessment: Sensitivity will be treated as the primary screening-safety metric, as missed eligible studies represent the principal risk of an assisted screening workflow. Specificity, precision, negative predictive value, inclusion agreement, and Cohen κ will be reported as secondary validation metrics.
- Data interpretation: Findings will be analyzed using predefined criteria to limit subjective interpretation. Any ambiguous data will be transparently documented and resolved by consensus.
- Conflict of interest management: Reviewers will declare potential conflicts of interest before beginning. Steps will be taken to ensure impartiality, including excluding conflicted reviewers from relevant decisions if necessary.
- Transparency and reproducibility: All review processes, from search strategies to data analysis methods, will be clearly documented. Standardized tools like PRISMA will enhance rigor and transparency.
- Pilot testing: Screening, data extraction, and analysis tools will be pilot-tested to address potential biases before formal use.

Traditional clinical risk of bias tools, such as ROBINS-I [38] and QUADAS-2 [39], are not tailored for evaluating studies involving LLMs in health care. To address this gap, we developed a domain-specific framework that synthesizes principles from established tools and literature:

- AI Fairness 360 Toolkit [40]: An open-source library by IBM Research designed to detect and mitigate bias in machine learning models, providing over 70 fairness metrics and 10 bias mitigation algorithms.
- PROBAST (Prediction model Risk Of Bias Assessment Tool) [41]: A tool for assessing the risk of bias and applicability of prediction model studies, focusing on participants, predictors, outcomes, and analysis.

Our framework captures key domains relevant to LLM evaluation, including dataset transparency, bias assessment methodology, mitigation strategies, and reporting transparency. These domains represent related but distinct aspects of study quality and are considered collectively to provide a comprehensive assessment.

It was iteratively refined through discussion within the research team to ensure clarity and applicability across diverse study designs. It will also be piloted on a subset of included studies to assess consistency, with further refinements made as necessary (Table 3).

**Table 3.** Tailored study quality assessment framework for medical large language model studies: domains and criteria^a^.

Domain	Assessment focus	Examples/operational guidance
1. Dataset transparency and representation	Clarity on dataset sources, demographics, and limitations. Evaluates whether training and evaluation data are described and representative of clinical diversity.	✔ Clearly names training/evaluation data (eg, PubMed, MIMIC-III^b^). ✔ Reports demographics, notes imbalances. ✖ No dataset source disclosed=High risk.
2. Bias assessment methods	Whether the study actively evaluates model bias using quantitative or qualitative methods. Studies that do not assess bias will be excluded according to eligibility criteria; therefore, this domain evaluates the robustness of bias assessment methods rather than their presence alone.	✔ Subgroup analysis by race/gender/SES^c^. ✔ Counterfactual or adversarial evaluation. ✖ Limited or poorly described bias assessment methods=High risk.
3. Bias mitigation techniques	Any method to reduce or control bias: in training, postprocessing, or model outputs.	✔ Appropriate mitigation strategy applied and evaluated (eg, debiasing algorithms, adversarial reweighting, RAG^d^, balanced training data) ✖ Mitigation strategy reported but poorly described, insufficiently justified, or not adequately evaluated=Moderate risk ✖ Study claims to mitigate bias but provides no clear method or evaluation, or mitigation is inadequately implemented=High risk If mitigation is not applicable to the study aim, this domain will be coded as “not applicable” rather than contributing to overall risk.
4. Explainability, transparency, and accountability	Does the study attempt to interpret model behavior and make it auditable?	✔ Uses SHAP^e^/LIME^f^ or other surrogate models. ✔ Applies transparency tools (eg, model cards, prompt disclosure). ✔ Acknowledges model uncertainty or auditability. ✖ Black-box outputs with no rationale=High risk.
5. Generalizability and evaluation setting	Contextual robustness: was the model tested across varied tasks, datasets, or populations?	✔ Uses external validation datasets. ✔ Reports performance across demographics. ✖ Tested only on narrow simulation=Moderate risk.
6. Ethical, regulatory, and clinical framing	Considers risks, benefits, alignment with AI ethics and medical governance. .	✔ Mentions national or international regulations or guidance. ✔ Discusses clinical safety, fairness, or misuse. ✖ No ethical or regulatory framing=High risk.

a Each domain will be rated as low, moderate, or high risk using predefined operational criteria. Low risk indicates comprehensive reporting and appropriate methodological implementation within the domain. Moderate risk indicates partial reporting or methodological limitations unlikely to invalidate study findings. High risk indicates substantial methodological limitations, inadequate reporting, or lack of reproducible evaluation methods likely to affect interpretability or reliability. Overall risk-of-bias judgments will be based on domain-level assessments. Studies will be classified as high risk if 2 or more domains are rated as high risk. This threshold was selected to ensure that studies with multiple methodological limitations are appropriately identified while avoiding overclassification based on a single domain. Final judgments will be confirmed through reviewer consensus. Risk levels are interpreted as follows. Low risk: fulfills low-risk standards in ≥4 domains with no domain rated high risk; Moderate risk: fulfills low-risk standards in 2 to 3 domains with no more than one domain rated high risk; High risk: two or more domains rated high risk. Studies that do not assess or report bias will be excluded according to the eligibility criteria.

b MIMIC-III: Medical Information Mart for Intensive Care III.

c SES: socioeconomic status.

d RAG: retrieval-augmented generation.

e SHAP: Shapley additive explanations.

f LIME: local interpretable model-agnostic explanations.

### Explainability Considerations

Explainability is a critical aspect of evaluating LLMs in health care [42]. It encompasses methods that provide human-understandable insights into model decisions. While tools like Shapley additive explanations [43] and local interpretable model-agnostic explanations [44] are commonly used, they represent just one category of explainability techniques. Recent surveys highlight a broader range of approaches, including gradient- and decomposition-based methods (eg, Integrated Gradients, Layer-wise Relevance Propagation), attention visualization and probing techniques, counterfactual and data influence analyses, concept attribution methods such as testing with concept activation vectors, and natural language or chain-of-thought explanations [45,46]. Structured documentation tools like model cards and datasheets also contribute to transparency. Transparency and accountability assessment may include reporting of prompt disclosure, rationale generation, uncertainty handling, auditability, evaluator blinding, and availability of prompts or outputs where applicable. Moreover, explainability is closely linked to interpretability (understanding the internal workings of the model) and accountability (ensuring mechanisms are in place to audit and trace model decisions). Together, these practices support the responsible evaluation and integration of LLMs in clinical settings [45].

In this review, we will assess whether included studies provide any form of interpretability or transparency regarding model behavior. We will examine whether the authors used any post hoc explanation techniques, model documentation approaches, or other methods to enhance model explainability or provide rationale attribution [43,44,46]. We will also assess whether these methods are applied systematically across outputs, whether their limitations are acknowledged, and whether the study includes any audit or traceability mechanism. Studies that present model outputs without any effort to explain or justify them will be considered high risk in this domain.

### Ethical Considerations

As a secondary analysis of published literature, ethical approval is not required.

### Dissemination

Results will be disseminated through peer-reviewed publications, academic conferences, and open-access repositories to inform responsible LLM deployment in health care. The results of this systematic review will be published in an open-access peer-reviewed journal and shared through presentations at relevant conferences focused on medical informatics, LLMs, and health care. The findings will be made available on open-access repositories, ensuring broad accessibility. Summary reports will be created to communicate key insights to nonacademic audiences.

Patient and Public Involvement

Although patients and the public were not directly involved in the design of this systematic review, the research team has engaged with standing patient and public involvement panels at Newcastle University through other ongoing projects. These panels have previously provided input on research priorities related to AI in health care, including concerns around fairness, trust, and transparency. Insights gained from those engagements informed the broader research context, particularly considerations of bias and ethical implications in clinical applications of LLMs. We intend to disseminate a plain-language summary of the review’s findings through relevant patient and public involvement networks to facilitate broader understanding and public dialogue.

### Reporting Standards

This protocol was developed in accordance with the PRISMA-P 2015 statement [37], and a completed PRISMA-P checklist is included as a Checklist 1. The completed systematic review will be reported in accordance with the PRISMA 2020 guidelines [47].

## Results

The phase 1 search was conducted on June 11, 2025, and yielded 15,976 records after deduplication. These records have undergone complete manual screening and assessment. The phase 2 search was conducted on June 8, 2026, and increased the total number of records to 41,143. Records from the updated search are undergoing LLM-assisted screening and eligibility assessment. Validation of the LLM-assisted workflow against the manually reviewed reference-standard dataset will be reported using sensitivity as the primary screening-safety metric, alongside specificity, precision, negative predictive value, inclusion agreement, and Cohen κ. The completed systematic review will report study selection using a PRISMA 2020 flow diagram. The review is expected to be submitted for publication in late November 2026, with subsequent publication dependent on the journal’s peer-review and editorial process.

## Discussion

### Expected Findings

This systematic review is expected to provide a comprehensive synthesis of current approaches to bias assessment and mitigation in medical LLMs and to highlight gaps in the consistency and depth of assessment.

While existing studies often acknowledge the presence of bias, the extent to which bias is systematically evaluated and addressed remains unclear. This review will therefore examine whether bias assessment is rigorously implemented and whether mitigation strategies are actively applied and evaluated across studies.

In addition, the review will compare reported mitigation approaches and their effectiveness, with the aim of identifying patterns in current practice and areas requiring further development.

The findings are expected to inform best practices for the assessment and management of bias in medical LLMs and may contribute to the development of more structured approaches or frameworks for evaluating bias in future research, supporting the development of more transparent, equitable, and clinically reliable AI systems.

### Strengths and Limitations

A key strength of this review is its comprehensive and interdisciplinary database coverage, incorporating both biomedical and computational research sources (eg, Embase, MEDLINE, PsycINFO, PubMed, ACL Anthology, ACM Digital Library, arXiv, medRxiv, and bioRxiv). The review also follows PRISMA-P guidelines and is preregistered on PROSPERO and OSF, enhancing transparency, reproducibility, and methodological rigor. In addition, the inclusion of gray literature enables the capture of emerging evidence that may not yet be available in peer-reviewed journals.

However, several limitations should be considered. Despite the inclusion of gray literature, publication bias may persist, as studies with neutral or negative findings may be underrepresented. Furthermore, heterogeneity in the definitions, measurement approaches, and reporting of bias across studies may limit comparability and preclude quantitative synthesis. Although the inclusion of preprint servers enables capture of the rapidly evolving literature on medical LLMs, studies available only as preprints have not undergone peer review and should therefore be interpreted with appropriate caution.

The LLM-assisted workflow will be validated against the completed manual review, but some risk of screening errors may remain. Relevant studies may be missed, particularly where reporting is unclear or incomplete. To reduce this risk, studies identified as eligible through the LLM-assisted workflow will undergo final review by at least 2 independent human reviewers before inclusion in the review. The completed review will report the final prompts, model versions, screening rules, validation results, and any workflow refinements made before application to the updated search results.

## Supplementary material

10.2196/91348 Multimedia Appendix 1 Full search strategy.

10.2196/91348 Multimedia Appendix 2 Search results and record counts.

10.2196/91348 Multimedia Appendix 3 Data extraction form template.

10.2196/91348 Checklist 1 PRISMA-P 2015 checklist.

## Abbreviations

CENTRAL: Cochrane Central Register of Controlled Trials
Epistemonikos: Evidence-Based Health Database
GRADE: Grading of Recommendations Assessment, Development and Evaluation
LLM: large language model
PCC: Population-Concept-Context
PRISMA: Preferred Reporting Items for Systematic Reviews and Meta-Analyses
PRISMA-P: Preferred Reporting Items for Systematic Reviews and Meta-Analyses Protocols
PROBAST: Prediction Model Risk of Bias Assessment Tool
PROSPERO: International Prospective Register of Systematic Reviews
RAG: retrieval-augmented generation
