# Supplementary material for: Exploring Bias in Medical Applications of Large Language Models: Protocol for a Systematic Review
Source: JMIR Res Protoc. 2026 Sep 10;15:e91348. doi: 10.2196/91348 (PMC13561442; doi:10.2196/91348)
Supplement: Supplementary file 1 [file resprot-v15-e91348-s001.docx]

**Supplementary Material 1: Full electronic search strategies**

#### 1. **EMBASE and MEDLINE (Ovid)**

**Platform:** Ovid
 **Date of last search:** 11 June 2025
 **Language limits:** English
 **Date limits:** 2017 onwards

((LLM* OR "Large Language Model*" OR LMM* OR MLLM OR "Vision-Language Models" OR "Audio-Language Models" OR "Speech Language Models" OR LVLM OR VLM OR GPT* OR BERT OR LaMDA OR PaLM OR LLaMA OR Claude OR Alpaca OR Falcon OR BLOOM OR "Generative AI" OR "Transformer-based model*" OR OPT OR Fairseq OR Deepseek OR Gemini OR Med-PaLM OR Radiology-LLaMA) AND (health* OR medic* OR clinic* OR patient* OR EHR OR physician* OR "Clinical Decision Support" OR "Medical AI" OR "Biomedical NLP" OR "Biomedical Natural Language Processing" OR BioNLP) AND (bias* OR prejudice* OR accuracy OR ethic* OR hallucination* OR fair* OR discrimination OR responsible OR "human value alignment" OR inequity OR disparit* OR equalit* OR "Algorithmic Bias" OR Explainability OR Transparency OR Accountability)).mp.

**Fields searched (.mp.):**
 Title, abstract, heading word, keyword heading word, floating subheading word, candidate term word, original title, device manufacturer, device trade name, drug trade name, drug manufacturer.

## 2. **ACM Digital Library**

**Platform:** ACM Digital Library
 **Date of last search:** 11 June 2025
 **Language limits:** English
 **Date limits:** 2017 onwards
 **Fields searched:** Title, Abstract

(Title:(LLM* OR "Large Language Model*" OR LMM* OR MLLM OR "Vision-Language Models" OR "Audio-Language Models" OR "Speech Language Models" OR LVLM OR VLM OR GPT* OR BERT OR LaMDA OR PaLM OR LLaMA OR Claude OR Alpaca OR Falcon OR BLOOM OR "Generative AI" OR "Transformer-based model*" OR OPT OR Fairseq OR Deepseek OR Gemini OR Med-PaLM OR Radiology-LLaMA) OR Abstract:(LLM* OR "Large Language Model*" OR LMM* OR MLLM OR "Vision-Language Models" OR "Audio-Language Models" OR "Speech Language Models" OR LVLM OR VLM OR GPT* OR BERT OR LaMDA OR PaLM OR LLaMA OR Claude OR Alpaca OR Falcon OR BLOOM OR "Generative AI" OR "Transformer-based model*" OR OPT OR Fairseq OR Deepseek OR Gemini OR Med-PaLM OR Radiology-LLaMA)) AND (Title:(health* OR medic* OR clinic* OR patient* OR EHR OR physician* OR "Clinical Decision Support" OR "Medical AI" OR "Biomedical NLP" OR "Biomedical Natural Language Processing" OR BioNLP) OR Abstract:(health* OR medic* OR clinic* OR patient* OR EHR OR physician* OR "Clinical Decision Support" OR "Medical AI" OR "Biomedical NLP" OR "Biomedical Natural Language Processing" OR BioNLP)) AND (Title:(bias* OR prejudice* OR accuracy OR ethic* OR hallucination* OR fair* OR discrimination OR responsible OR "human value alignment" OR inequity OR disparit* OR equalit* OR "Algorithmic Bias" OR Explainability OR Transparency OR Accountability) OR Abstract:(bias* OR prejudice* OR accuracy OR ethic* OR hallucination* OR fair* OR discrimination OR responsible OR "human value alignment" OR inequity OR disparit* OR equalit* OR "Algorithmic Bias" OR Explainability OR Transparency OR Accountability))

## 3. **Web of Science (Preprints / Core Collection)**

**Platform:** Web of Science
 **Date of last search:** 11 June 2025
 **Language limits:** English
 **Date limits:** 2017 onwards
 **Field searched:** Topic (TS)

TS=((LLM* OR "Large Language Model*" OR LMM* OR MLLM OR "Vision-Language Models" OR "Audio-Language Models" OR "Speech Language Models" OR LVLM OR VLM OR GPT* OR BERT OR LaMDA OR PaLM OR LLaMA OR Claude OR Alpaca OR Falcon OR BLOOM OR "Generative AI" OR "Transformer-based model*" OR OPT OR Fairseq OR Deepseek OR Gemini OR Med-PaLM OR Radiology-LLaMA) AND (health* OR medic* OR clinic* OR patient* OR EHR OR physician* OR "Clinical Decision Support" OR "Medical AI" OR "Biomedical NLP" OR "Biomedical Natural Language Processing" OR BioNLP) AND (bias* OR prejudice* OR accuracy OR ethic* OR hallucination* OR fair* OR discrimination OR responsible OR "human value alignment" OR inequity OR disparit* OR equalit* OR "Algorithmic Bias" OR Explainability OR Transparency OR Accountability))

## 4. **PsycINFO (Ovid)**

**Platform:** Ovid
 **Database:** PsycINFO
 **Date of last search:** 11 June 2025
 **Language limits:** English
 **Date limits:** 2017 onwards

((LLM* OR "Large Language Model*" OR LMM* OR MLLM OR "Vision-Language Models" OR "Audio-Language Models" OR "Speech Language Models" OR LVLM OR VLM OR GPT* OR BERT OR LaMDA OR PaLM OR LLaMA OR Claude OR Alpaca OR Falcon OR BLOOM OR "Generative AI" OR "Transformer-based model*" OR OPT OR Fairseq OR Deepseek OR Gemini OR Med-PaLM OR Radiology-LLaMA) AND (health* OR medic* OR clinic* OR patient* OR EHR OR physician* OR "Clinical Decision Support" OR "Medical AI" OR "Biomedical NLP" OR "Biomedical Natural Language Processing" OR BioNLP) AND (bias* OR prejudice* OR accuracy OR ethic* OR hallucination* OR fair* OR discrimination OR responsible OR "human value alignment" OR inequity OR disparit* OR equalit* OR "Algorithmic Bias" OR Explainability OR Transparency OR Accountability)).mp.

## 5. **ProQuest (Health & Medical / Dissertations / Databases)**

**Platform:** ProQuest
 **Date of last search:** 11 June 2025
 **Language limits:** English
 **Date limits:** 2017 onwards

(LLM* OR "Large Language Model*" OR LMM* OR MLLM OR "Vision-Language Models" OR "Audio-Language Models" OR "Speech Language Models" OR LVLM OR VLM OR GPT* OR BERT OR LaMDA OR PaLM OR LLaMA OR Claude OR Alpaca OR Falcon OR BLOOM OR "Generative AI" OR "Transformer-based model*" OR OPT OR Fairseq OR Deepseek OR Gemini OR Med-PaLM OR Radiology-LLaMA) AND (health* OR medic* OR clinic* OR patient* OR EHR OR physician* OR "Clinical Decision Support" OR "Medical AI" OR "Biomedical NLP" OR "Biomedical Natural Language Processing" OR BioNLP) AND (bias* OR prejudice* OR accuracy OR ethic* OR hallucination* OR fair* OR discrimination OR responsible OR "human value alignment" OR inequity OR disparit* OR equalit* OR "Algorithmic Bias" OR Explainability OR Transparency OR Accountability
