# Supplementary material for: Exploring Bias in Medical Applications of Large Language Models: Protocol for a Systematic Review
Source: JMIR Res Protoc. 2026 Sep 10;15:e91348. doi: 10.2196/91348 (PMC13561442; doi:10.2196/91348)
Supplement: Supplementary file 2 [file resprot-v15-e91348-s002.docx]

**Supplementary Material 2: Search results and record counts**

A comprehensive systematic search was conducted across multiple bibliographic and preprint databases to identify studies examining bias in medical applications of large language models (LLMs). Searches were designed to capture literature from both healthcare and artificial intelligence research domains. The original search was conducted on 11 June 2025. An updated search using the same information sources and search strategy was conducted on 8 June 2026 to capture the rapidly expanding evidence base.

## **Databases searched and records retrieved**

### **Table S1. Records retrieved by database (original search, 11 June 2025)**

| **Database** | **Platform** | **Records retrieved** |
| --- | --- | --- |
| EMBASE | Ovid | 11,290 |
| MEDLINE | Ovid | 4,283 |
| PsycINFO | Ovid | 870 |
| ACM Digital Library | ACM | 592 |
| Web of Science (Preprints) | Clarivate | 1,694 |
| ProQuest (Health & Medical collections) | ProQuest | 5,243 |
| **Total** | — | **23,972** |

All retrieved records were imported into Rayyan ([https://www.rayyan.ai](https://www.rayyan.ai/)) for reference management and deduplication. Automated duplicate detection within Rayyan identified 7,996 duplicate records, which were removed prior to screening. Following deduplication, 15,976 unique records remained for title and abstract screening.

**Table S2. Records retrieved by database (updated search, 8 June 2026)**

| **Database** | **Platform** | **Records retrieved** |
| --- | --- | --- |
| EMBASE, MEDLINE & PsycINFO | Ovid | 28,561 |
| ACM Digital Library | ACM | 982 |
| Web of Science (Preprints) | Clarivate | 24661 |
| ProQuest (Health & Medical collections) | ProQuest | 5,236 |
| **Total** | — | **59,440** |

All retrieved records were imported into the LLM-assisted screening workflow used for this review, where automated deduplication was performed. Duplicate detection identified 18,297 duplicate records, leaving 41,143 unique records for title and abstract screening.

Notes on reporting:

- Record counts reflect the results obtained directly from each database at the time of the original and updated searches.
- Preliminary pilot searches conducted during search strategy development yielded different interim totals; only results from the final standardised searches conducted on 11 June 2025 and the updated search conducted on 8 June 2026 are reported.
- The original search underwent complete manual screening, full-text assessment, data extraction, and quality assessment. Screening of records identified through the updated search is currently ongoing using the LLM-assisted workflow described in the protocol.
- As screening of the updated search is ongoing, title and abstract screening, full-text assessment, and final study inclusion will be reported using a PRISMA 2020 flow diagram in the completed systematic review.

Software and tools used:

- The original search was managed using Rayyan for reference management, deduplication, and screening.
- The updated search was managed using the LLM-assisted screening workflow described in the protocol, which performed automated deduplication prior to screening.
- Full electronic search strategies for all databases are reported in Supplementary Material 1.
