# Supplementary material for: Exploring Bias in Medical Applications of Large Language Models: Protocol for a Systematic Review
Source: JMIR Res Protoc. 2026 Sep 10;15:e91348. doi: 10.2196/91348 (PMC13561442; doi:10.2196/91348)
Supplement: Supplementary file 3 [file resprot-v15-e91348-s003.docx]

**Data Extraction Form Template**

| **Section** | **Data Item** | **Data Format / Options** | **Notes** |
| --- | --- | --- | --- |
| **1. Study Identification** | Title | Text | Full study title |
|  | Authors | Text | First author, et al. |
|  | Year of Publication | YYYY | - |
|  | Country of Corresponding Author | Text | - |
|  | Journal / Conference | Text | - |
| **2. Study Design** | Study Type | Dropdown: Experimental, Observational, Benchmarking, Simulation/Vignette-based | Define methodology |
|  | Sample Size | Number | If applicable |
|  | Primary Objective | Text | - |
| **3. LLM Model Details** | Model Type | Dropdown: encoder-only, decoder-only, encoder-decoder | Describe name of the model: GPT, BERT, PaLM, LLaMA, Claude, Alpaca, Falcon, BLOOM, Other |
|  | Model Size (# of Parameters) | Number | - |
|  | Hyperparameters Reported? | Yes/No | Top-p, temperature, fine-tuning process |
|  | General or Domain-specific? | Yes/No |  |
|  | Continued Fine-tuning Procedure? | Yes/No | Describe if applicable |
|  | Prompt Engineering Used? | Yes/No | Describe if applicable |
| **4. Medical Application** | Domain | Dropdown: Clinical Decision Support, Medical Documentation, Patient Interaction, Biomedical Research, Education | Multiple selections allowed |
|  | Evaluation Setting | Dropdown: Real-world clinical, Simulated, Dataset Benchmark | - |
| **5. Bias Assessment Methods** | Bias Detection Method | Dropdown: Dataset Audit, Counterfactual Testing, Adversarial Evaluation, Fairness Metrics, Not Reported | - |
|  | Fairness Metrics Reported? | Yes/No | Demographic Parity, Equalized Odds, Other |
|  | Demographic Groups Evaluated | Dropdown: Race, Gender, Age, Socioeconomic, None | - |
| **6. Bias Mitigation Strategies** | Bias Mitigation Applied? | Yes/No | - |
|  | Bias Mitigation Method | Dropdown: Data Augmentation, Algorithmic Debiasing, Adversarial Training, Retrieval-Augmented Generation (RAG), Post-hoc Filtering | Multiple selections allowed |
|  | Effectiveness of Mitigation Reported? | Yes/No | If yes, summarize key findings |
| **7. Transparency & Explainability** | Explainability Method Used? | Dropdown: SHAP, LIME, Other, None | - |
|  | Justification for Model Outputs Provided? | Yes/No | - |
| **8. Generalizability & External Validation** | External Validation Performed? | Yes/No | - |
|  | Dataset Split for Validation? | Dropdown: Train-Test Split, Cross-validation, None | - |
| **9. Ethical & Clinical Implications** | Ethical Considerations Discussed? | Yes/No | Bias risks, regulatory concerns, fairness principles |
|  | Potential Harms Identified? | Yes/No | If yes, describe |
|  | Recommendations for Responsible AI? | Yes/No | If yes, summarize |
| **10. Risk of Bias Assessment** | Overall Risk of Bias | Dropdown: Low, Moderate, High | Based on framework criteria |
|  | Reviewer Comments | Text | Additional observations |
